# Appropriate NH_4_^+^: NO_3_^−^ ratio improves low light tolerance of mini Chinese cabbage seedlings

**DOI:** 10.1186/s12870-017-0976-8

**Published:** 2017-01-23

**Authors:** Linli Hu, Weibiao Liao, Mohammed Mujitaba Dawuda, Jihua Yu, Jian Lv

**Affiliations:** 1 College of Horticulture, Gansu Agricultural University, No. 1 Yingmen Village, Anning District, Lanzhou, 730070 People’s Republic of China; 2 Department of Horticulture, FoA, University for Development Studies, P. O. Box TL 1882, Tamale, Ghana

**Keywords:** Chlorophyll fluorescence imaging, Calvin cycle, Relative gene expression, Low light fluence, Ammonium: nitrate ratio

## Abstract

**Background:**

In northwest of China, mini Chinese cabbage (*Brassica pekinensis*) is highly valued by consumers, and is widely cultivated during winter in solar-greenhouses where low light (LL) fluence (between 85 and 150 μmol m^−2^ s^−1^ in day) is a major abiotic stress factor limiting plant growth and crop productivity. The mechanisms with which various NH_4_^+^: NO_3_^−^ ratios affected growth and photosynthesis of mini Chinese cabbage under normal (200 μmol m^−2^ s^−1^) and low (100 μmol m^−2^ s^−1^) light conditions was investigated. The four solutions with different ratios of NH_4_^+^: NO_3_^−^ applied were 0:100, 10:90, 15:85 and 25:75 with the set up in a glasshouse in hydroponic culture. The most appropriate NH_4_^+^: NO_3_^−^ ratio that improved the tolerance of mini Chinese cabbage seedlings to LL was found in our current study.

**Results:**

Under low light, the application of NH_4_^+^: NO_3_^−^ (10:90) significantly stimulated growth compared to only NO_3_^−^ by increasing leaf area, canopy spread, biomass accumulation, and net photosynthetic rate. The increase in net photosynthetic rate was associated with an increase in: 1) maximum and effective quantum yield of PSII; 2) activities of Calvin cycle enzymes; and 3) levels of mRNA relative expression of several genes involved in Calvin cycle. In addition, glucose, fructose, sucrose, starch and total carbohydrate, which are the products of CO_2_ assimilation, accumulated most in the cabbage leaves that were supplied with NH_4_^+^: NO_3_^−^ (10:90) under LL condition. Low light reduced the carbohydrate: nitrogen (C: N) ratio while the application of NH_4_^+^: NO_3_^−^ (10:90) alleviated the negative effect of LL on C: N ratio mainly by increasing total carbohydrate contents.

**Conclusions:**

The application of NH_4_^+^:NO_3_^−^ (10:90) increased *rbcL*, *rbcS*, *FBA*, *FBPase* and *TK* expression and/or activities, enhanced photosynthesis, carbohydrate accumulation and improved the tolerance of mini Chinese cabbage seedlings to LL. The results of this study would provide theoretical basis and technical guidance for mini Chinese cabbage production. In practical production, the ratio of NH_4_^+^:NO_3_^−^ should be adjusted with respect to light fluence for successful growing of mini Chinese cabbage.

**Electronic supplementary material:**

The online version of this article (doi:10.1186/s12870-017-0976-8) contains supplementary material, which is available to authorized users.

## Background

In northwest of China, mini Chinese cabbage (*Brassica pekinensis*) is highly valued by consumers, and is widely cultivated during winter in solar-greenhouses where low light (LL) fluence (between 85 and 150 μmol m^−2^ s^−1^ in day) is a major abiotic stress factor limiting plant growth and crop productivity. The exposure of most plants to LL condition affects metabolism in various ways including the alteration of enzyme activity (e.g. transketolase, superoxide dismutase, catalase) and disruption of transcription [1, 2]. Low light fluence also inhibited violaxanthin de-epoxidase, causing failure of the protective xanthophyll cycle [3]. The alleviation role of 5-aminolevulinic acid and calcium in cucumber under LL condition was reported [4, 5]. Therefore, understanding the genetic and biochemical processes that regulate LL tolerance is a vital area of research in plant biology.

Ammonium (NH_4_^+^) and nitrate (NO_3_^−^) improved plants tolerance to environmental stress, including low light stress [6], drought [7], salinity [8], alkalinity [9], disease [10], heavy metal toxicity [11], higher CO_2_ concentration [12], and ultraviolet radiation [13]. In addition, NH_4_^+^ and NO_3_^−^ have different effects on physiological and biochemical processes including photorespiration, photosynthesis, nutrient absorption and nitrogen metabolism [14]. Cruz et al. [12] demonstrated that increasing NH_4_^+^ significantly increased photosynthetic acclimation to elevated CO_2_ during the early growth stage of cassava. However, the negative effect of NH_4_^+^ on photosynthesis was also observed at the later growth stages.

Improving photosynthesis is vital to maintaining sufficient dry biomass accumulation, especially in plants subjected to LL condition. The total CO_2_ assimilation rate is limited by light intensity, temperature, CO_2_ diffusion (stomatal conductance), enzyme activity (Rubisco), substrate availability (RuBP regeneration), and respiratory CO_2_ release [15]. For increasing plant yield, identifying limiting points in photosynthetic process is the central issue. Calvin cycle contains 11 different enzymes which catalyze 13 reactions in the three phases of carboxylation, reduction and regeneration. It is initiated by the enzyme ribulose-1, 5-bisphosphate carboxylase oxygenase (Rubisco) which catalyses the carboxylation of the CO_2_ acceptor molecule, ribulose-1, 5-bisphosphate (RuBP). The 3-phosphoglycerate (3-PGA) formed in carboxylation stage is then used to form the 3-phosphate glyceraldehyde (GAP) and dihydroxyacetone phosphate (DHAP), through two reactions that consume ATP and NADPH. The glyceraldehyde-3-phosphate dehydrogenase (GAPDH) plays an important role in the second reaction. The regenerative phase of the cycle involves a series of reactions. These reactions convert GAP and DHAP into the CO_2_ acceptor molecule, RuBP. The fructose-1, 6-bisphosphatase (FBPase), fructose-1, 6-bisphosphate aldolase (FBA) and transketolase (TK) play the vital roles in these reactions. The GAP produced in the Calvin cycle mostly remains within the cycle to regenerate RuBP, while only few exits from the cycle and are utilized to synthesis sucrose and starch [16]. The mechanism involved in photosynthesis with different nitrogen forms has been reviewed by Guo et al. [14], and includes changes in stomatal density and mesophyll conductance, alteration in photosynthetic enzyme activities, and changes in accumulation of photosynthetic outcome. As a consequence, photosynthesis is limited mainly by light intensity, light use efficiency and CO_2_ assimilation capacity among other factors.

As demonstrated by Lu et al. [17], total replacement of NO_3_^−^ by NH_4_^+^ induced a reduction of stomatal conductance and a decreased dry weight. Moreover, nitrogen in the form of NO_3_^−^ alone or NH_4_NO_3_ resulted in a greater dry weight gain in tobacco than when NH_4_^+^ was applied alone. Although there have been several studies on altered expression of genes coding for Calvin cycle enzymes after exposure to exogenous substance or under environmental stress in cucumber [2, 18], the consequences for NH_4_^+^- induced enhancement of photosynthesis under LL condition has not been extensively explored. However, the application of moderate NH_4_^+^ was recently found to have enhanced the LL tolerance of mini Chinese cabbage seedlings in an earlier experiment [6]. To understand the underlying mechanism of moderate NH_4_^+^- induced promotion of net photosynthetic rate (Pn), investigations are needed to conclusively confirm that the presence of NH_4_^+^ in the nutrient solution promotes photosynthesis. In this study, we reported the mechanisms with which moderate NH_4_^+^: NO_3_^−^ improved the tolerance of mini Chinese cabbage seedlings to LL condition in the greenhouse.

## Results

### Plant growth and biomass accumulation

**Fig. 1:**
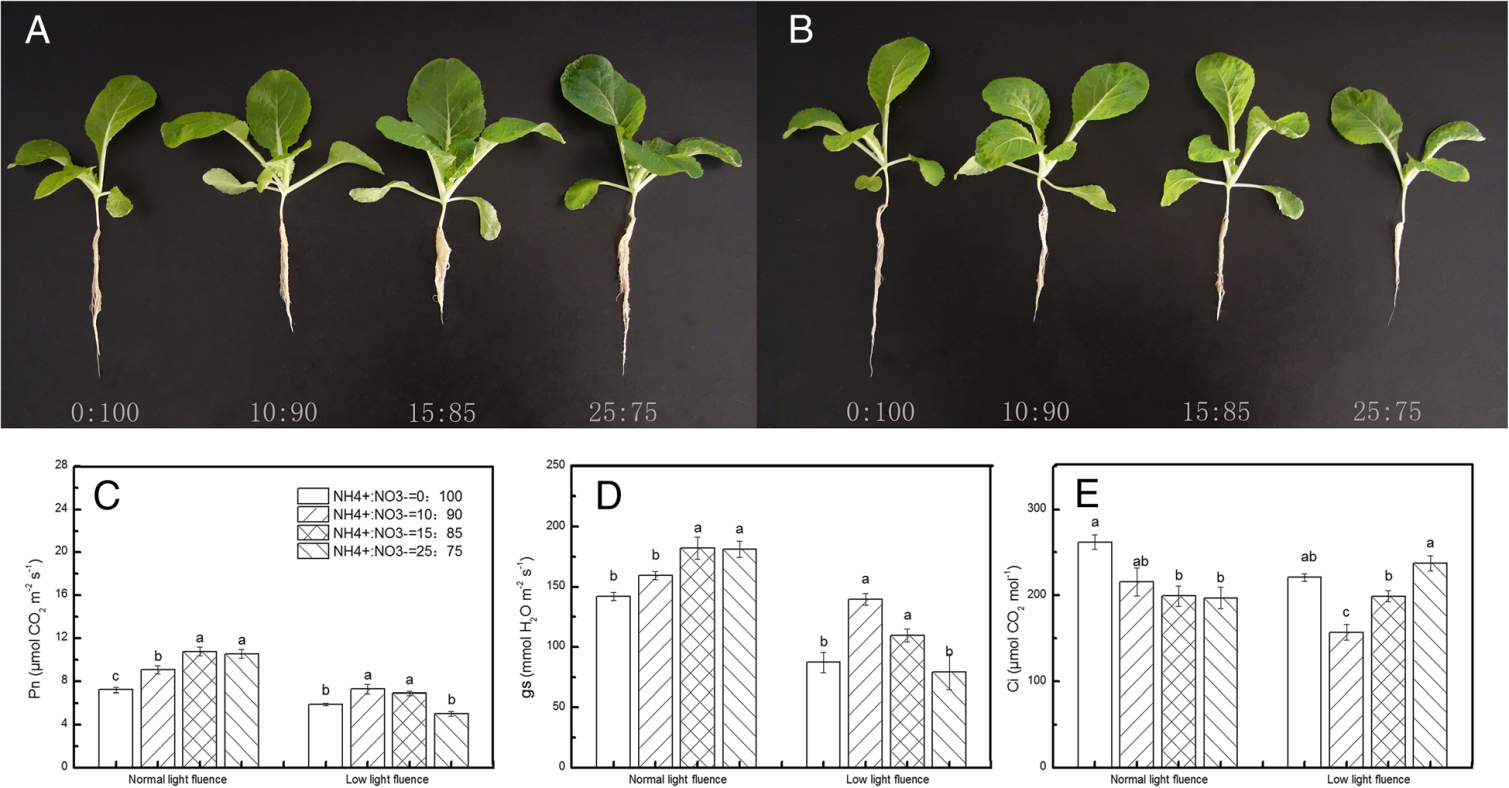
Effects of NH_4_^+^:NO_3_^−^ on growth and gas exchange parameters at 9 days after treatment in mini Chinese cabbage leaves under normal (200 μmol m^−2^ s^−1^) and low (100 μmol m^−2^ s^−1^) light fluences. Mini Chinese cabbage seedlings treated with NH_4_^+^:NO_3_^−^ ratios for 15 days under normal light fluence (**a**) and low light fluence (**b**). The photosynthetic parameters Pn (**c**), gs (**d**) and Ci (**e**) in mini Chinese cabbage seedling leaves were measured at 9 days after NH_4_^+^: NO_3_^−^ and light fluence treatment. Data represent means of three replicates. Bars indicate the SE. Significant differences (*P* < 0.05) between treatments are indicated by different letters, for each light condition

Under normal light condition, when compared with the control (NH_4_^+^:NO_3_^−^ = 0:100), leaf area, canopy spread, fresh weight, dry weight and chlorophyll content in plants fed with NH_4_^+^:NO_3_^−^ (15:85) were significantly increased by 33.8, 30.5, 77.9, 72.9 and 33.7%, respectively (Table 1). The plants under LL stress grew slowly and had decreased total leaf area and canopy spread. However, leaf number was neither influenced by light condition nor NH_4_^+^: NO_3_^−^ applications, suggesting that it is likely to be developmentally controlled. The biomass under LL condition also decreased as a consequence of reduced total leaf area (Table 1; Fig. 1 a, b).

**Table 1:** Effects of ammonium: nitrate on leaf area, canopy spread, leaf number, fresh weight, dry weight and total chlorophyll content in mini Chinese cabbage under normal (200 μmol m^−2^ s^−1^) and low (100 μmol m^−2^ s^−1^) light conditions

Light fluence	NH_4_^+^:NO_3_^−^	Leaf area (cm^2^ plant^−1^)	Canopy spread (cm^2^ plant^−1^)	Leaf number (# plant-1)	Fresh weight (g plant^−1^)	Dry weight (g plant^−1^)	Chlorophyll (SPAD)
Normal light fluence	0:100	66.86 ± 3.46 b^a^	94.55 ± 1.83 c	5.27 ± 0.10 a	4.409 ± 0.147 c	1.153 ± 0.014 c	21.27 ± 0.58 b
	10:90	68.72 ± 4.93 b	113.06 ± 1.15 b	5.43 ± 0.09 a	6.696 ± 0.079 ab	1.638 ± 0.012 b	25.57 ± 0.33 a
	15:85	89.44 ± 4.71 a	123.38 ± 1.25 a	5.47 ± 0.10 a	7.842 ± 0.308 a	1.994 ± 0.043 a	28.43 ± 0.61 a
	25:75	88.71 ± 1.72 a	111.95 ± 3.29 b	5.40 ± 0.10 a	6.582 ± 0.413 b	1.604 ± 0.033 b	26.93 ± 1.26 a
Low light fluence	0:100	48.24 ± 1.43 b	57.16 ± 1.24 bc	5.00 ± 0.07 a	2.243 ± 0.078 bc	0.617 ± 0.009 bc	23.87 ± 0.50 c
	10:90	58.08 ± 2.64 a	65.65 ± 1.64 a	5.13 ± 0.06 a	3.497 ± 0.080 a	0.842 ± 0.022 a	28.63 ± 0.68 a
	15:85	54.95 ± 1.71 ab	63.21 ± 1.12 ab	4.93 ± 0.08 a	2.502 ± 0.065 b	0.672 ± 0.033 b	26.87 ± 0.18 ab
	25:75	48.64 ± 1.13 b	55.56 ± 1.64 c	4.83 ± 0.12 a	1.991 ± 0.068 c	0.553 ± 0.013 c	25.90 ± 0.50 bc

^a^Data are expressed as means ± SE (*n* = 3 with 20 plants per replication). Means followed by the different letters are significantly different according to Tukey’s test (*P* < 0.05), in each of the two light conditions

NH_4_^+^: NO_3_^−^ (10:90) application improved plant growth as indicated by increased total leaf area, canopy spread, fresh and dry weight of plants under LL (Table 1; Fig. 1 a, b). Moreover, application of NH_4_^+^:NO_3_^−^ (10:90) under LL condition led to a significant increase in total chlorophyll content, almost reaching the levels of plants under normal light. In contrast, NH_4_^+^: NO_3_^−^ (0:100 and 25:75) application under LL condition resulted in 42 and 45% reduction in fresh weight compared with NH_4_^+^:NO_3_^−^ (10:90) application, respectively (Table 1). As shown in Table 1, all the growth parameters and chlorophyll content were almost recovered to those under normal light condition levels when NH_4_^+^:NO_3_^−^ (10:90) was applied to the LL fluence treated plants.

To further investigate the interdependence of the responses to NH_4_^+^:NO_3_^−^ ratios and light conditions, principal component analyses of growth parameters and chlorophyll content was carried out. The results showed that the two principal components were selected as the total contribution rate of them was greater than 95% (Table 2). The first principal components, which may be the most effective coefficient and index, included the traits of leaf area, canopy spread, leaf number, fresh weight and dry weight. The second principal component only included chlorophyll content, which was also effective coefficient and index (Additional file 1: Table S3). From the ranking of treatments based on general scores in Table 2, we observed: 1) NH_4_^+^: NO_3_^−^ (15:85) application was beneficial for plant growth under normal light condition; 2) LL inhibited plant growth; 3) NH_4_^+^: NO_3_^−^ (10:90) application enhanced LL tolerance of mini Chinese cabbage seedlings.

**Table 2:** Principal component analysis of tested traits of mini Chinese cabbage under four NH_4_^+^:NO_3_^−^ ratios and normal (200 μmol m^−2^ s^−1^) and low (100 μmol m^−2^ s^−1^) light fluences

Treatments		Principal component scores		General scores	Ranking
Light fluence	NH_4_^+^:NO_3_^−^	1	2		
Normal light fluence	0:100	0.091	−0.329	−0.238	4
	10:90	1.502	−0.075	1.427	3
	15:85	2.691	0.115	2.806	1
	25:75	1.892	0.033	1.925	2
Low light fluence	0:100	−1.829	−0.103	−1.932	7
	10:90	−0.751	0.201	−0.55	5
	15:85	−1.501	0.104	−1.397	6
	25:75	−2.094	0.053	−2.041	8
Eigenvalues		4.87	0.98	-	-
Comulative (%)		81.17	97.50	-	-

### Gas exchange and chlorophyll fluorescence parameters

**Fig. 2:**
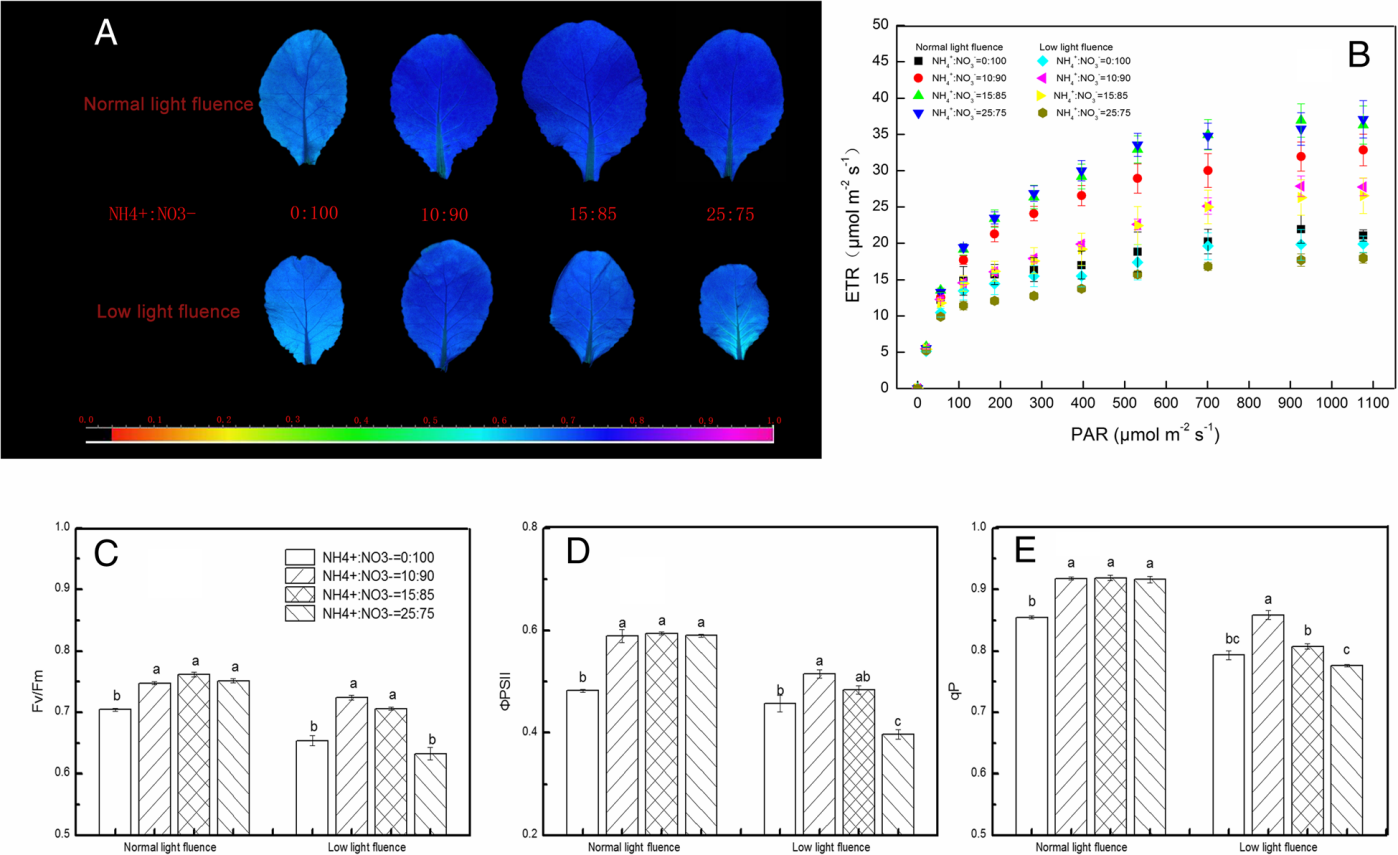
Effects of NH_4_^+^:NO_3_^−^ on chlorophyll fluorescence parameters at 9 days after treatment in mini Chinese cabbage leaves under normal (200 μmol m^−2^ s^−1^) and low (100 μmol m^−2^ s^−1^) light fluences. (**a**) Images of Fv/Fm. The fluorescence images of the Fv/Fm are given in false colors that represent the absolute values of the ratio ranged from 0 (*black*) to 1.0 (*purple*). Three seedlings in each treatment were measured. (**b**) ETR, (**c**) Fv/Fm, (**d**) ΦPSII and (**e**) qP. Data represent means of three area of interest from three leaves. Bars indicate the SE (*n* = 3). Significant differences (*P* < 0.05) between treatments are indicated by different letters, for each light condition

The net photosynthetic rate (Pn), stomatal conductance (gs) and intercellular CO_2_ concentration (Ci) directly reflect plant photosynthetic capacity, and whether the limiting factor of photosynthesis is stomatal or not can be judged by these gas exchange parameters. The reduction of light fluence was the most determinant factor for all investigated parameters (Figs. 1 and 2). As shown in Fig. 1 c, in normal light fluence plants, the highest Pn was observed in 15:85 (NH_4_^+^:NO_3_^−^) and the lowest in 0:100 (NH_4_^+^:NO_3_^−^). The plants supplied with NH_4_^+^:NO_3_^−^ (10:90) under LL condition showed a significantly higher Pn than those fertilized solely with NO_3_^−^. Furthermore, decreased Pn in LL treated plants was partially recovered by NH_4_^+^:NO_3_^−^ (10:90) application (Fig. 1 c). The negative effect of sole NO_3_^−^ was observed on gs (Fig. 1 d). However, Ci increased in plants treated with only NO_3_^−^ and NH_4_^+^:NO_3_^−^ (25:75) application, but decreased in plants treated with NH_4_^+^:NO_3_^−^ (10:90) under LL condition (Fig. 1 e).

Under our growth conditions, low light fluence caused visible stress symptoms and significantly decreased the maximum quantum yield of PSII (Fv/Fm) in plants fertilized with NH_4_^+^:NO_3_^−^ (0:100) and NH_4_^+^:NO_3_^−^ (25:75). However, NH_4_^+^:NO_3_^−^ (10:90) application resulted in increased Fv/Fm under LL condition (Fig. 2 a, c). In normal light plants, NH_4_^+^:NO_3_^−^ (0:100) significantly decreased Fv/Fm, effective quantum yield of PSII (ΦPSII) and photochemical quenching (qP), whereas Fv/Fm, ΦPSII and qP did not respond to NH_4_^+^:NO_3_^−^ ratio in the presence of NH_4_^+^ (Fig. 2 c, d, e). In LL plants, NH_4_^+^:NO_3_^−^ (0:100) application caused reduced ΦPSII, and this reduction of ΦPSII was recovered by 10:90 or 15:85 NH_4_^+^:NO_3_^−^ application (Fig. 2 d). Moreover, treatments with NH_4_^+^:NO_3_^−^ (0:100, 15:85 and 25:75) significantly reduced qP, but NH_4_^+^:NO_3_^−^ (10:90) application recovered qP to the level of qP in the plants under normal light (Fig. 2 e). ETR gradually increased and tended to be steady with the increase of photosynthetic active radiation (PAR). Low light caused significant reduction in ETR. NH_4_^+^:NO_3_^−^ (10:90) application alleviated LL stress and enhanced the ETR. Moreover, the ETRs of plants treated with NH_4_^+^:NO_3_^−^ (10:90) under LL fluence were significant higher than those treated with NH_4_^+^:NO_3_^−^ (0:100) under normal light fluence (Fig. 2 b).

### Activation of Calvin cycle enzymes

**Fig. 3:**
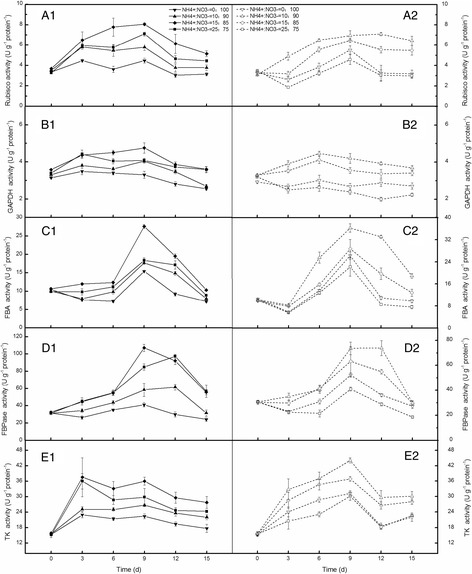
Activities of main enzymes in Calvin cycle in mini Chinese cabbage seedling leaves at 0, 3, 6, 9, 12, and 15 days after treatments. Capital letters A to E represent Rubisco, GAPDH, FBA, FBPase and TK activities, respectively. Letters are followed by 1 and 2 that represent normal (200 μmol m^−2^ s^−1^) and low (100 μmol m^−2^ s^−1^) light conditions. Vertical bars represent mean ± SE value from three independent replicates (*n* = 3)

A sharp increase in Rubisco activity was observed in plants treated with NH_4_^+^:NO_3_^−^ (15:85), which reached its highest levels on day 9, and then decreased in the following days and then remained unchanged (Fig. 3 A1). However, the Rubisco activity in plants treated with NH_4_^+^:NO_3_^−^ (0:100) increased gently only during the first 3 days, and then remained relatively constant, indicating that the addition of NH_4_^+^ in nutrient solution enhanced Rubisco activity in cabbage leaves. The Rubisco activity of plants treated with NH_4_^+^:NO_3_^−^ (0:100 and 25:75) in LL condition decreased during the first 3 days, then gradually increased and reached its highest levels on day 9, decreased in the following days. However, when NH_4_^+^:NO_3_^−^ (10:90) was added to the solution, the activity of Rubisco gradually increased until steady level (Fig. 3 A2). The activities of GAPDH (Fig. 3 B1, B2) in the leaves were either unaltered or slightly increased in the NH_4_^+^:NO_3_^−^ (15:85) and NH_4_^+^:NO_3_^−^ (10:90) treated seedlings, indicating that light fluence and NH_4_^+^:NO_3_^−^ slightly influenced GAPDH activity. The activities of FBA in plants with NH_4_^+^:NO_3_^−^ (10:90) under LL condition remained high on day 6, 9, 12 and 15, and exhibited significant difference compared with the plants fed with the other three NH_4_^+^:NO_3_^−^ ratios (Fig. 3 C1, C2). The activities of FBPase decreased in LL treated plants but the NH_4_^+^:NO_3_^−^ (10:90) application alleviated the reduction (Fig. 3 D1, D2). The highest activities of TK in normal light condition was attained on day 3 but on day 9 in LL condition (Fig. 3 E1, E2). The increase of TK activity in NH_4_^+^:NO_3_^−^ (15:85) treated plants was much steeper than that in NH_4_^+^:NO_3_^−^ (0:100) treated plants, particularly from day 0 to day 3 (Fig. 3 E1). For example, the TK activity of 15:85 (NH_4_^+^:NO_3_^−^) treatments was 63% greater than that of the control on day 3 (Fig. 3 E1). Similarly, the positive effects on TK activity of NH_4_^+^:NO_3_^−^ (10:90) were also largely enhanced in LL plants (Fig. 3 E2).

### Relative expression of Calvin cycle genes

**Fig. 4:**
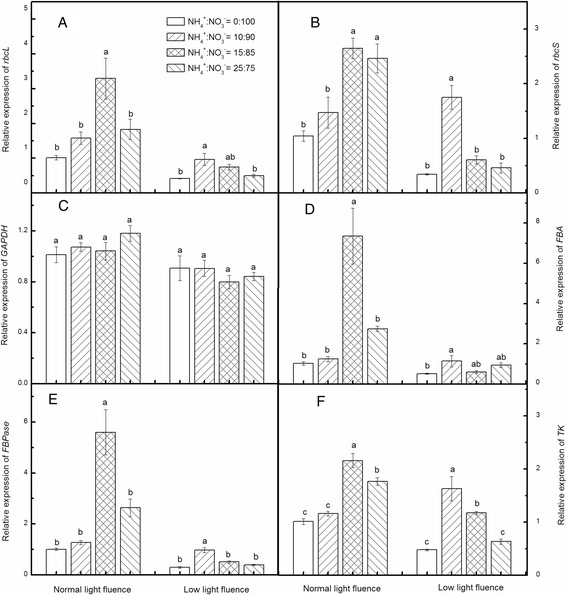
Effects of NH_4_^+^:NO_3_^−^ on the relative expression level of genes involved in Calvin cycle under normal (200 μmol m^−2^ s^−1^) and low (100 μmol m^−2^ s^−1^) light conditions. Leaf samples were harvested at 9 days after NH_4_^+^:NO_3_^−^ ratios and light intensities treatment. Capital letters **a-f** represent expression levels of *rbcL*, *rbcS*, *GAPDH*, *FBA*, *FBPase* and *TK*, respectively. Data are the means of three replicates with SEs shown by vertical bars. Means followed by the different letters are significantly different according to Tukey’s test (*P* < 0.05), for each light condition

The results showed that the relative expression levels of *rbcL* (Fig. 4 a), *rbcS* (Fig. 4 b), *GAPDH* (Fig. 4 c), *FBA* (Fig. 4 d), *FBPase* (Fig. 4 e) and *TK* (Fig. 4 f) were up-regulated in NH_4_^+^-treated seedlings grown under normal light condition. Interestingly, expression levels of all the six genes except *GAPDH* increased by 2.6–7.3 -fold in leaves of plants treated with NH_4_^+^:NO_3_^−^ (15:85) (Fig. 4). NH_4_^+^:NO_3_^−^ and light fluence had no significant effect on the expression of *GAPDH* (Fig. 4 c). The transcript levels of these genes were down-regulated by low light, and when plants grown under LL condition were supplied with NH_4_^+^:NO_3_^−^ (10:90), the relative expressions of *rbcL*, *FBA* and *FBPase* were completely recovered to the control level (Fig. 4 a, d, e) while the relative expression of *rbcS* and *TK* was above the control level (Fig. 4 b, f).

### Carbohydrate, total nitrogen and C: N ratio

**Fig. 5:**
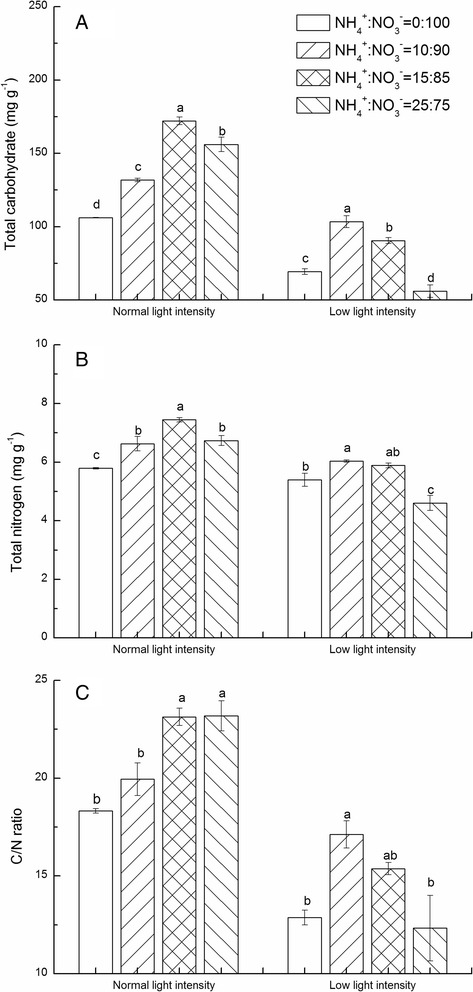
Effects of NH_4_^+^:NO_3_^−^ on total carbohydrate (**a**), total nitrogen (**b**) and C/N ratio (**c**) in mini Chinese cabbage leaves under normal (200 μmol m^−2^ s^−1^) and low (100 μmol m^−2^ s^−1^) light conditions. Data represent means of three replicates. Bars indicate the SE (*n* = 3). Significant differences (*P* < 0.05) between treatments are indicated by different letters, for each light condition

The effect of NH_4_^+^:NO_3_^−^ on carbohydrate metabolism under different light fluences was evaluated. Similar to the changes in Pn, the glucose, fructose, sucrose and starch levels all increased in the leaves of ammonium-treated plants under normal light condition. Moreover, the maximum values of glucose, fructose and starch levels were reached in plants treated with NH_4_^+^:NO_3_^−^ (15:85), and the maximum value of sucrose level was reached in plants treated with NH_4_^+^:NO_3_^−^ (25:75), but no significant difference was observed between NH_4_^+^:NO_3_^−^ (15:85) and NH_4_^+^:NO_3_^−^ (25:75) treatments (Table 3). The decrease in the glucose, fructose, sucrose and starch levels in plants grown under LL condition was partially restored by NH_4_^+^:NO_3_^−^ (10:90) application (Table 3). As shown in Fig. 5 a, the content of total carbohydrate increased and then decreased with the increase of ammonium concentration in solutions under two light conditions, whereas the highest values were observed in NH_4_^+^:NO_3_^−^ (15:85) under normal light fluence and in NH_4_^+^:NO_3_^−^ (10:90) under LL fluence. Low light decreased the total carbohydrate content, but the NH_4_^+^:NO_3_^−^ (10:90) application alleviated the negative effects of LL on total carbohydrate content (Fig. 5 a).

**Table 3:** Effects of NH_4_^+^:NO_3_^−^ on levels of glucose, fructose, sucrose and starch at 15 days after NH_4_^+^:NO_3_^−^ treatment in mini Chinese cabbage under normal (200 μmol m^−2^ s^−1^) and low (100 μmol m^−2^ s^−1^) light conditions

Light fluence	NH_4_^+^:NO_3_^−^	Glucose (%)	Fructose (%)	Sucrose (%)	Starch (%)
Normal light fluence	0:100	4.38 ± 0.26 b^a^	3.53 ± 0.21 c	0.23 ± 0.03 b	2.47 ± 0.06 c
	10:90	5.39 ± 0.17 ab	4.21 ± 0.39 bc	0.30 ± 0.02 b	3.28 ± 0.21 b
	15:85	6.02 ± 0.46 a	6.32 ± 0.16 a	0.53 ± 0.06 a	4.34 ± 0.24 a
	25:75	5.73 ± 0.25 a	5.25 ± 0.46 ab	0.54 ± 0.03 a	4.08 ± 0.19 a
Low light fluence	0:100	2.49 ± 0.14 ab	2.72 ± 0.09 bc	0.09 ± 0.02 b	1.64 ± 0.10 ab
	10:90	3.35 ± 0.20 a	3.98 ± 0.12 a	0.23 ± 0.03 a	2.78 ± 0.43 a
	15:85	3.05 ± 0.25 a	3.11 ± 0.14 b	0.15 ± 0.02 b	2.75 ± 0.35 a
	25:75	1.76 ± 0.32 b	2.41 ± 0.18 c	0.07 ± 0.01 b	1.36 ± 0.12 b

^a^Data are expressed as means ± SE (*n* = 3). Means followed by the different letters are significantly different according to Tukey’s test (*P* < 0.05), in each of the two light conditions

Higher levels of nitrogen accumulated in shoot of plants treated with NH_4_^+^:NO_3_^−^ (15:85) than in those treated with NH_4_^+^:NO_3_^−^ (0:100) under normal light condition (Fig. 5 b). However, the highest level of total nitrogen was observed in NH_4_^+^:NO_3_^−^ (10:90) treatment under LL condition (Fig. 5 b). Results presented in Fig. 5 c show that the C: N ratio reached the highest value in NH_4_^+^:NO_3_^−^ (25:75) treated plants, and no significant difference was observed between NH_4_^+^:NO_3_^−^ (15:85) applied plants. Low light decreased the C: N ratio, but the application of NH_4_^+^:NO_3_^−^ (10:90) suppressed the negative effects of LL on C: N ratio (Fig. 5 c).

## Discussion

Photosynthesis is the main process by which plants produce and accumulate dry matter. The importance of water, light, nutrient and CO_2_ in this process cannot be over emphasized. In the present study, we found that moderate NH_4_^+^: NO_3_^−^ application enhanced vegetative characteristics, while non-ammonium or higher ammonium application resulted in reduced growth under normal and low light conditions. This observation suggests that NH_4_^+^ is actively involved in the promotion of plant growth. Furthermore, in order to obtain the maximum biomass, less concentration of ammonium was needed in LL condition than in normal light condition. This was probably because low light reduced carbon metabolism of plants and less carbohydrate were accumulated in the tissues of the plants. Thus, less photosynthate was transported from the shoot to the root in LL condition, and thus the energy used for ammonium assimilation in plant root was low. Therefore, higher NH_4_^+^ concentration in LL condition was not completely assimilated by plant root, and then accumulated in plant cells causing ammonium toxicity. Sakakibara et al. [19] reported that inorganic nitrogen (NH_4_^+^ or NO_3_^−^) was a substrate for nitrogen assimilation and also functions as a signal triggering widespread changes in gene expression that modulate metabolism and development. They also demonstrated that a large research project that focused on nitrate action in gene expression of *Arabidopsis* has provided the view of the extent of nitrate-dependent regulatory genes, including nitrogen metabolism, carbon metabolism and cytokinin responses. We also observed that nitrogen metabolism of plants decreased under LL condition, but the extent of the decrease of nitrogen metabolism was less than carbon metabolism, causing reduced C: N ratio. However, the application of 10% NH_4_^+^ in solutions enhanced C: N ratio mainly by increasing the total carbohydrate contents. Therefore, appropriate NH_4_^+^ concentration in solution was beneficial for plants to maintain the balance of carbon and nitrogen metabolism. These results demonstrate that the level of NH_4_^+^ is a rate-limiting factor for plant growth, and supplying appropriate NH_4_^+^ levels for plants according to the light fluence is an effective way to promote plant growth and improve vegetable yield.

### Calvin cycle is the main rate-limiting factor of CO_2_ assimilation

Nitrogen can significantly affect the performance of the three main processes of photosynthesis: stomatal control of CO_2_ supply, thylakoid electron transport (light reaction), and Calvin cycle (dark reaction) [20, 21]. Our results indicate that the Pn rate of NH_4_^+^-treated plants was higher than those of sole NO_3_^−^-treated plants. Low light fluence caused a decrease in Pn per unit leaf area at 9 days after treatment under NH_4_^+^:NO_3_^−^ (0:100) application. However, the negative influence of LL was not observed in plants treated with NH_4_^+^: NO_3_^−^ (10:90). The addition of appropriate NH_4_^+^ is beneficial for improved photosynthesis in LL condition. This finding was consistent with Golvano et al. [22], who demonstrated that NH_4_^+^-fed plants had higher protein content and increased activity of photosynthetic enzymes compared with NO_3_^−^-fed plants. Frantz et al. [23] also reported that the inhibition of Pn was caused by ammonium toxicity. Claussen and Lenz [24] found that NH_4_^+^ accumulation in leaves led to uncoupling of the electron transport form photophosphorylation in chloroplasts, which consequently led to decreased photosynthetic rate. In our study, we also observed that the reduction of photosynthesis in plants fertilized with sole NO_3_^−^ was accompanied by decreased chlorophyll content, but this did not occur in plants treated with NH_4_^+^: NO_3_^−^ (25:75). This, therefore, suggests that the reduced photosynthesis is not the result of reduced light-harvesting capacity but possibly as a consequence of decreased gas conductance. It could also be due to the activities of Calvin cycle enzymes or negative feedback regulation by accumulated carbon metabolites after sole NO_3_^−^ treatment or higher NH_4_^+^ concentration (25%) treatment. We also observed that plants fed with only NO_3_^−^ had significantly lower gs but higher Ci, thus, reduced gas conductance as a major factor for reduced CO_2_ assimilation was excluded. Finally, the decreased Pn in plants treated with only NO_3_^−^ was consistent with reduced contents of glucose, fructose, sucrose and starch, making it unlikely that negative feedback regulation by accumulated carbon metabolites resulted in reduced photosynthetic rates. Therefore, our results suggest that the net photosynthetic rate of plant treated by NH_4_^+^: NO_3_^−^ and light fluence was probably limited by the Calvin cycle.

The chlorophyll fluorescence imaging is a helpful measurement to investigate several aspects of photosynthesis. This is because it reflects changes in thylakoid membrane organization and function and inhibition of photosynthesis and oxygen evolution through interactions with components of PSII [25, 26]. In the present study, the sole application of NO_3_^−^ significantly reduced ETR and Fv/Fm in normal light condition. Low light fluence obviously reduced ETR and Fv/Fm, while the addition of 10 and 15% NH_4_^+^ inhibited the negative effect of LL on ETR and Fv/Fm in different levels. As described by Krause and Weis [27], damage to component of thylakoid membranes, especially those of PSII, and inhibition of energy transfers from antenna molecules to reaction centers can result in lower Fv/Fm. In the chloroplast ultrastructure of mini Chinese cabbage, the degree of granal stacking increased in NH_4_^+^:NO_3_^−^ (10:90) treated plants under LL condition [6]. Similarly, Bi et al. [2] reported a decline in Fv/Fm, ΦPSII after exposure to low temperature and low light intensity in transgenic cucumber plants. In our study, in normal light plants, the addition of NH_4_^+^ increased ΦPSII and qP, but the sole NO_3_^−^ decreased the ΦPSII and qP; while ΦPSII in LL plants significantly decreased in higher NH_4_^+^ (25%) treatment. Higher ammonium concentration (25%) applied to mini Chinese cabbage seedlings under LL condition greatly degraded the grana lamella and decreased the light-captured area [6]. The significant decrease in ΦPSII was mainly attributed to the decrease in degree of granal stacking, which is deemed as ‘down regulation’ of light energy absorbed area. In NH_4_^+^:NO_3_^−^ (10:90) treated plants under low light, the increase in qP was attributed to the increase in the consumption rate of reductants and ATP generated from non-cyclic electron transport caused by enhanced carboxylation rate.

### NH_4_^+^:NO_3_^−^ and light fluence regulate activities of several Calvin cycle enzymes and expression of genes coding these enzymes

The photosynthesis rate is limited by the carboxylation reaction of Rubisco and the capacity of RuBP regeneration [28]. For instance, the capacity of RuBP regeneration determines the photosynthetic rate under low irradiance or high CO_2_ concentration. Analysis of antisense plants with decreased activity of Rubisco showed that the control coefficient on photosynthesis varied from 0.1 to 0.9 depending on the experimental and growth conditions such as light intensity and CO_2_ concentration [29], suggesting that Rubisco activity does not always dominate the photosynthesis rate and that other rate-limiting steps exist.

Our results showed that NH_4_^+^: NO_3_^−^ and light fluence affected light capture system and the dark reaction - Calvin cycle. Moreover, Calvin cycle is the main rate-limiting factor of CO_2_ assimilation. NH_4_^+^: NO_3_^−^ regulates photosynthetic capacity, to a large extent, by affecting the capacity of Rubisco carboxylation and RuBP regeneration. The application of NH_4_^+^: NO_3_^−^ (15:85) to the plants under normal light significantly enhanced activities of Rubisco, GAPDH, FBA, FBPase and TK, and the results was similar to the application of 10:90 (NH_4_^+^: NO_3_^−^) in LL plants. Raab and Terry [30, 31] demonstrated that the Rubisco content of leaf in NH_4_^+^ supplied sugar beet plants was significantly higher than those in NO_3_^−^supplied plants, and similar results were obtained by Guo et al. [32] when they conducted their experiment with rice plants. In the present study, the relative expressions of *rbcS*, *rbcL*, *FBA*, *FBPase* and *TK* were up regulated in NH_4_^+^-treated plants under normal light. Low light fluence inhibited the expression of these genes in different levels, but *rbcL*, *FBA* and *FBPase* were partially restored. The expression of *rbcS* and *TK* were above the control level in NH_4_^+^: NO_3_^−^ (10:90) treated plants. This result is similar to what was observed in rice leaves during ontogeny [33, 34]. As described by Wingler et al. [35], low light intensity results in reduced expression of light-dependent genes and the disappearance of photosynthetic proteins. Several studies have illustrated that up regulation of genes involved in Calvin cycle leads to increased Pn and enhanced vegetative growth, while reduced expression of these genes results in stunted plant growth [18, 36]. We also observed that the relative expression of *FBA* in plants under LL fed with NH_4_^+^: NO_3_^−^ (25:75) was higher than those fed with NH_4_^+^: NO_3_^−^ (15:85), whereas the enzyme activity of FBA was reversed. Changes in enzyme activity and gene expression under NH_4_^+^: NO_3_^−^ and light fluence conditions are not always positively correlated, suggesting the possibility of further regulatory mechanisms. The results were consistent with Oelze et al. [37], who reported that transcript abundance is poorly linked to de novo protein synthesis due to profound regulation at the level of translation. In general, effects of NH_4_^+^: NO_3_^−^ and light fluence on transcript levels and enzymes’ activities suggest that NH_4_^+^: NO_3_^−^ and light fluence play important role in the synthesis and activities of enzymes involved in Calvin cycle.

RuBP regeneration capacity depends on both photosynthetic electron transport chain and the enzymes downstream of Rubisco in the Calvin cycle [38]. In this study, we observed that while the addition of appropriate NH_4_^+^ concentration promoted photosynthetic electron transport and expression of genes encoding the Calvin cycle enzymes required for RuBP regeneration, the application of sole NO_3_^−^ inhibited these. Among the examined enzymes involved in Calvin cycle, Rubisco is a vital enzyme that catalyzes the carboxylation of the CO_2_ acceptor molecule, RuBP, GAPDH catalysed the conversion of 1, 3 - diphosphoglyceric acid into GAP, while FBPase catalyzes a rate-limiting step in the Calvin cycle and carbohydrate metabolism [39]. The Rubisco, GAPDH and FBPase are activated by light fluence, usually in two ways: by changing the microenvironment and by producing effector. FBA and TK are not controlled by iron - thioredoxin, but they are controlled by carbon fixation of photosynthesis to a greater extent [40, 41]. For example, a decreased in FBA level resulted in reduced RuBP content, and then inhibited photosynthesis and growth of transgenic potato [42]. In a previous study, when TK activity decreased from 20 to 40%, it led to a significant reduction in RuBP regeneration and significantly inhibited photosynthetic rate of plants [43]. In our current study, the activities of these enzymes in plants fed with only NO_3_^−^ or higher NH_4_^+^ decreased in 3 days after exposure to LL fluence and then gradually increased afterwards. NH_4_^+^: NO_3_^−^ (10:90) application alleviated the negative effect of LL on the activities of these Calvin cycle enzymes. As demonstrated by Rigano et al. [44], ammonium assimilation required photosynthates imported through the phloem, causing a transitory decrease in the concentration of ATP, along with noticeable variations in glucose-6 -Pconcentration, a permanent decrease in free glucose concentration, an increase in respiratory oxygen consumption. The consumption of photosynthates drives the Calvin cycling process. Thus, the activities of Calvin cycling enzymes were activated. Rigano et al. [44] also demonstrated that less ammonium was utilized in plants under dark condition than in illuminated plants. Therefore, the addition of moderate NH_4_^+^ in LL plants is beneficial for activating Calvin cycling enzymes. The application of NH_4_^+^: NO_3_^−^ (10:90) enhanced activity of Calvin cycle enzymes in LL fluence, and this was probably due to the fact that the absorbed NH_4_^+^changed the microenvironment of chloroplast, or the NH_4_^+^ assimilation activated the Calvin cycle.

## Conclusions

Our results have shown that appropriate NH_4_^+^ concentration in nutrient solution under LL condition significantly increased the Fv/Fm, the activities of Calvin cycle enzymes, the relative expression of these genes, the levels of glucose, fructose, sucrose, starch, total carbohydrate and nitrogen, and C: N ratio in mini Chinese cabbage seedlings in comparison with those from plants treated solely with NO_3_^−^ or higher amounts of NH_4_^+^. The enhancement of photosynthesis and LL tolerance in mini Chinese cabbage seedlings supplied with NH_4_^+^:NO_3_^−^ (10:90) is largely attributed to the increase in *rbcL*, *rbcS*, *FBA*, *FBPase* and *TK* expression (and/or activities).

## Methods

### Materials and experimental design

The seeds of Mini Chinese cabbage (*Brassica pekinensis* cv. “Jinwa no. 2”) obtained from Gansu Academy of Agricultural Sciences, Lanzhou, China, were germinated on moist filter paper in the dark at 25 °C for 16 h, and then sown in clean quartz sand medium and fertilized with half-strength Hoagland’s nutrient solution [45] once a day. The seedlings were raised in a modern climate-controlled greenhouse with a photoperiod of 12 h, temperature of 23 ± 2/13 ± 2 °C (day/night), and light fluence about 200 μmol m^−2^ s^−1^. When the second fully expanded leaves of the seedlings appeared, groups of 20 uniform seedlings were transplanted into a container (38 cm × 28 cm × 12 cm) filled with 6 L NH_4_^+^: NO_3_^−^ treated solutions and aerated at 4 h intervals. These plants were separately placed in normal light fluence (200 μmol m^−2^ s^−1^) and low light fluence (100 μmol m^−2^ s^−1^) and supplied with one of the following NH_4_^+^: NO_3_^−^ ratios: 0:100, 10:90, 15:85 and 25:75. The two light conditions were regulated by sunshade nets and incandescent lamps.

The composition of the nutrition solution used was as follows: 5 mmol L^−1^ N, using Ca(NO_3_)_2_ · 4H_2_O, KNO_3_ as NO_3_^−^–N and using (NH_4_)_2_SO_4_ as NH_4_^+^–N, 1 mmol L^−1^ P as KH_2_PO_4_, 3 mmol L^−1^ K as KNO_3_, K_2_SO_4_ and KH_2_PO_4_, 1.5 mmol L^−1^ Ca as Ca(NO_3_)_2_ · 4H_2_O, CaSO_4_ · 2H_2_O and CaCl_2_, 2 mmol L^−1^ Mg as MgSO_4_ · 7H_2_O, plus standard micronutrients referred to Hoagland and Arnon [45]. All elements which plant essentially need remain the same concentration in 4 NH_4_^+^:NO_3_^−^ treatments (Additional file 2: Table S1). Nitrification inhibitor (DCD, 7 μmol L^−1^) was supplied to every container. The pH of nutrient solutions in each container was adjusted to 6.5–7.0 by adding 0.1 mol L^−1^ HCl or NaOH solution once a day. The 6-L nutrient solution was changed once a week to avoid depletion effects. The containers, each with twenty seedlings, were arranged in a completely randomized design in the greenhouse.

### Measurement of canopy spread, leaf area and biomass

Fourteen days after application of the treatments, the leaf spread of the cabbage seedlings was considered to be elliptical and therefore was computed as follows: canopy spread = π × A × B / 4, where A is the longest spread of two opposite leaves and B is the shortest spread of two opposite leaves when placed gently on a flat surface. Total leaf area was measured with leaf area analyzer (YMJ-C, Tuopu Instruments Inc. China).

When seedlings were harvested, the fresh weight of the whole plant was recorded. Subsequently, all grouped samples were kept in an oven first at 105 °C for 15 min and then at 80 °C until constant weight. The dry weight of all samples were determined and recorded using a digital balance.

### Measurement of gas exchange parameters and total chlorophyll content

Net photosynthetic rate (Pn), stomatal conductance (gs), and intercellular CO_2_ concentration (Ci) were measured from the second young fully expanded leaves using a portable photosynthetic system (CIRAS-2, PP System, UK). The photosynthetic photon flux density (200 μmol m^−2^ s^−1^), ambient CO_2_ concentration (380 μmol mol^−1^), leaf temperature (25 °C) and relative humidity (75%) were maintained throughout the measurements.

The measured leaves were labeled and total chlorophyll content was determined with SPAD meter (leaf chlorophyll meter, SPAD-502 plus, Tuopu Instruments Inc. China). The leaf samples were then frozen in liquid nitrogen and stored at −80 °C for determining the activities and relative expression levels of the genes coding for enzymes of the Calvin cycle.

### Imaging of chlorophyll fluorescence

Nine days after treatments started, chlorophyll fluorescence induction parameters were investigated using an Imaging-PAM Chlorophyll Fluorometer (Walz, Effeltrich, Germany). Before measurement, the plant leaves were kept in darkness for 30 min to allow all reaction centers to open. With the Imaging- PAM, Fs, Fo (steady chlorophyll fluorescence of light-adapted leaves, minimum fluorescence yield of the dark-adapted leaves, respectively) and Fm′, Fm (maximum fluorescence yield of the light & dark-adapted leaves, respectively) were obtained with the application of a saturation pulse. The maximum quantum yield of PSII (Fv /Fm = (Fm- Fo)/ Fm) and effective quantum yield of PSII (ФPSII) (ФPSII = (Fm’ - Fs) / Fm’) was calculated according to Genty et al. [46]. Light- adapted minimal fluorescence (Fo’) was measured when the actinic light was turned off in the presence of far-red light. Photochemical quenching (qP) was calculated according to qP = (Fm’- Fs) / (Fm’-Fo’) [47]. The electron transport rates (ETR) at a given actinic irradiance [Photosynthetic active radiation (PAR) = 0, 21, 56, 111, 186, 281, 396, 531, 701, 926, 1076 μmol m^−2^ s^−1^] were determined according to White and Critchley [48].

### Measurement of Calvin cycle enzymes activity

The second leaves from the top of plants were sampled at 0, 3, 6, 9, 12 and 15 days after treatments to determine enzyme activity. The activities of Ribulose-1, 5-bisphosphate carboxylase/oxygenase (Rubisco, EC 4.1.1.39), Glyceraldehyde-phosphate dehydrogenase (GAPDH, EC 1.2.1.12), Fructose-1, 6-bisphosphatase (FBPase, EC 3.13.11), Fructose-1, 6-bisphosphate aldolase (FBA, EC 4.1.2.13), and Transketolase (TK, EC 2.2.1.1) were determined with ELISA kit (Shanghai Yanji Biological Technology Ltd., China) and the extraction method of these enzymes were as described by Rao and Terry [49] with minor modifications. The frozen leaf samples (0.5 g) were ground to a fine powder in liquid N_2_ with a mortar and pestle and transferred into a centrifuge tube, then extracted in pre-chilled extraction buffer (5 ml). The enzyme extraction solution was centrifuged for 15 min at 12,000× *g* and 4 °C. The supernatant was used for Calvin cycle enzymes activity assay. Subsequently, the activities of Calvin cycle enzymes were determined with Microplate Absorbance Reader (BioTek ELX800, USA) in absorbance at 450 nm according to the manufacturer’s instruction.

The protein concentration of each enzyme extraction solution was measured by the method of Bradford [50]. The results were expressed as U g^−1^ of protein.

### Total RNA extraction and gene expression analysis

Total RNA was extracted using RNAiso Plus (TaKaRa D9108A) according to the supplier’s instruction. The relative mRNA expression of Calvin cycle enzyme genes in mini Chinese cabbage plants were analyzed by real time quantitative RT-PCR using a SYBR® Green QPCR MIX QPS-201 T (TOYOBO), according to the manufacturer’s instructions. The mini Chinese cabbage *actin* gene (GenBank accession No. JN120480.1) was used as an internal control. The primers were designed and synthesized by Sangon Biotech Co., Ltd. (Shanghai, China). On the basis of nucleotide, the primers for rubisco large subunit gene (*rbc*L), rubisco small subunit gene (*rbc*S) glyceraldehyde-3-phosphate dehydrogenase (*GAPDH*), fructose-1, 6- bisphosphatase (*FBPase*), fructose-1, 6-bisphosphate aldolase (*FBA*), transketolase (*TK*), and *actin* genes were designed and used for amplification. Gene bank accession numbers of the sequences used to design the primers were provided in the Additional file 3: Table S2.

Each real-time PCR reaction system was performed in a final volume of 20 μl on a Real-Time PCR Detection System (ABI stepone plus, USA) using the following program: 5 min at 95 °C followed by 40 cycles of 10 s at 95 °C and 30 s at 60 °C with data collection at the annealing step. After the 40 cycles, we included the dissociation /melting curve stage with 15 s at 95 °C, 60 s at 60 °C, and 15 s at 95 °C. The relative quantification of mRNA levels is based on the method of Livak and Schmittgen [51]. The threshold cycle value (Ct) of *actin* was subtracted from that of the target gene to obtain a ΔCt value. The Ct value of the control sample in experiment was subtracted from the ΔCt value to obtain a ΔΔCt value. The expression level relative to the control for each sample was expressed as 2^-ΔΔCt^. All the samples were analyzed three times.

### Carbohydrate, total nitrogen and C: N ratio analysis

The carbohydrate (glucose, fructose, sucrose and starch) levels were measured using anthrone-sulfuric method, as described by Halhoul and Kleinberg [52] with a little modification. The total carbohydrate content was determined by glucose, fructose, sucrose plus starch content. Total nitrogen levels in the shoot tissues were measured using the Kjeldahl method as described by Knowles and Ries [53] with a little modification. The carbohydrate: nitrogen (C: N) ratio was derived using the respective values of carbohydrate and nitrogen.

### Data analysis

Tukey’s test was used for testing the significance of means difference between treatments by using the SPSS 16.0. Using the R correlation matrix method, principal component analysis of growth parameters was carried out with SPSS 16.0. All figures were created by Origin ver. 8.5 (OriginLab Institute Inc. USA).

## Additional files

Additional file 1: Table S3. Principal components loading matrix of tested traits of mini Chinese cabbage under four ammonium: nitrate ratios and two light fluences. (DOCX 16 kb)
Additional file 2: Table S1. The concentrations of salts (mmol L^−1^) used to prepare macronutrient solutions at NH_4_^+^: NO_3_^−^ ratios of 0: 100, 10:90, 15:85 and 25:75. (DOCX 16 kb)
Additional file 3: Table S2. Primer sequences, annealing temperature and Genebank accession number of the *rbcL*, *rbcS*, *GAPDH*, *FBPase*, *FBA*, *TK* and *actin* gene sequences. (DOCX 18 kb)

## Abbreviations

*C*i: Intercellular CO_2_ concentration
ETR: Electron transport rate
FBA: Fructose-1, 6-bisphosphate aldolase (EC 4.1.2.13)
FBPase: Fructose-1, 6-bisphosphatase (EC 3.13.11)
Fv/Fm: Maximum quantum yield of PSII
GAPDH: Glyceraldehyde-phosphate dehydrogenase (EC 1.2.1.12)
gs: Stomatal conductance
Pn: Net photosynthetic rate
qP: Photochemical quenching
*rbcL*: Rubisco large subunit gene
*rbcS*: Rubisco small subunit gene
Rubisco: Ribulose-1, 5-bisphosphate carboxylase/oxygenase (EC 4.1.1.39)
TK: Transketolase (EC 2.2.1.1)
ΦPSII: Effective quantum yield of PSII
