# Supplementary material for: Appropriate NH4+: NO3− ratio improves low light tolerance of mini Chinese cabbage seedlings
Source: BMC Plant Biol. 2017 Jan 23;17:22. doi: 10.1186/s12870-017-0976-8 (PMC5259974; doi:10.1186/s12870-017-0976-8)
Supplement: Additional file 1: Table S3. — Principal components loading matrix of tested traits of mini Chinese cabbage under four ammonium: nitrate ratios and two light fluences. (DOCX 16 kb) [file 12870_2017_976_MOESM1_ESM.docx]

**Table S3.** Principal components loading matrix of tested traits of mini Chinese cabbage under four ammonium: nitrate ratios and two light fluences.

| Items | Principal components | |
| --- | --- | --- |
|  | 1 | 2 |
| Leaf area | 0.958^a^ | 0.060 |
| Growth range | 0.985 | -0.146 |
| Leaf number | 0.966 | -0.149 |
| Fresh weight | 0.997 | 0.003 |
| Dry weight | 0.995 | -0.022 |
| Chlorophyll content | 0.257 | 0.966 |

^a^ Data present the correlation coefficients of principal components and tested traits.
