# Supplementary material for: Appropriate NH4+: NO3− ratio improves low light tolerance of mini Chinese cabbage seedlings
Source: BMC Plant Biol. 2017 Jan 23;17:22. doi: 10.1186/s12870-017-0976-8 (PMC5259974; doi:10.1186/s12870-017-0976-8)
Supplement: Additional file 2: Table S1. — The concentrations of salts (mmol L−1) used to prepare macronutrient solutions at NH4 +: NO3 − ratios of 0: 100, 10:90, 15:85 and 25:75. (DOCX 16 kb) [file 12870_2017_976_MOESM2_ESM.docx]

**Table S1** The concentrations of salts (mmol L^-1^) used to prepare macronutrient solutions at NH_4_^+^: NO_3_^-^ ratios of 0: 100, 10:90, 15:85 and 25:75

| NH_4_^+^:NO_3_^-^ | Salts in the nutrient solutions (mmol L^-1^) | | | | | | | |
| --- | --- | --- | --- | --- | --- | --- | --- | --- |
|  | Ca(NO_3_)_2_.4H_2_O | KNO_3_ | (NH_4_)_2_SO_4_ | K_2_SO_4_ | CaSO_4_.2H_2_O | CaCl_2_ | KH_2_PO_4_ | MgSO_4_.7H_2_O |
| 0:100 | 1.5 | 2 | 0 | 0 | 0 | 0 | 1 | 2 |
| 10:90 | 1.3 | 1.9 | 0.25 | 0.05 | 0.1 | 0.1 | 1 | 2 |
| 15:85 | 1.2 | 1.85 | 0.375 | 0.075 | 0.15 | 0.15 | 1 | 2 |
| 25:75 | 1 | 1.75 | 0.625 | 0.125 | 0.25 | 0.25 | 1 | 2 |
