# Supplementary material for: Appropriate NH4+: NO3− ratio improves low light tolerance of mini Chinese cabbage seedlings
Source: BMC Plant Biol. 2017 Jan 23;17:22. doi: 10.1186/s12870-017-0976-8 (PMC5259974; doi:10.1186/s12870-017-0976-8)
Supplement: Additional file 3: Table S2. — Primer sequences, annealing temperature and Genebank accession number of the rbcL, rbcS, GAPDH, FBPase, FBA, TK and actin gene sequences. (DOCX 18 kb) [file 12870_2017_976_MOESM3_ESM.docx]

| Gene symbol | Primer sequences | Annealing temperature (℃) | Genebank accession number |
| --- | --- | --- | --- |
| *rbcL* | F: 5'-ACAACTGTGTGGACCGATGG-3' | 58.4 | GQ184364 |
|  | R: 5'-CTTCTCCTGGAACGGGCTC-3' | 59.4 |  |
| *rbcS* | F: 5'-ACCCTCTCTTACCTTCCTGACC-3' | 58.3 | KJ508094 |
|  | R: 5'-GCTTGTAGGCGATGAAACTGA-3' | 58.1 |  |
| *GAPDH* | F: 5'-GGACTGGAGAGGTGGAAGAGC-3' | 60.1 | XM_009125769 |
|  | R: 5'-AACAACTGAAACATCAACGGTG-3' | 57.5 |  |
| *FBPase* | F: 5'-GTCGGTGATTTTCATAGGACTTTG-3' | 60.0 | KJ508096 |
|  | R: 5'-CTGATGTATCTCGGTCGGTTGG-3' | 61.8 |  |
| *FBA* | F: 5'-TCGCTAACGCCGCATACATC-3' | 62.1 | KJ544564 |
|  | R: 5'-ACCGCTGAGGCAAGGGAGA-3' | 62.7 |  |
| *TK* | F: 5'-GGGAAGCAAGACACCTGGACAT-3' | 62.7 | XM_009140569 |
|  | R: 5'-GCTAAACCAACAGCATTCGCAA-3' | 63.0 |  |
| *actin* | F: 5'-CCAGGAATCGCTGACCGTAT-3' | 59.9 | JN120480 |
|  | R: 5'-CTGTTGGAAAGTGCTGAGGGA-3' | 60.1 |  |

**Table S2.** Primer sequences, annealing temperature and Genebank accession number of the *rbcL*, *rbcS*, *GAPDH, FBPase, FBA, TK* and *actin* gene sequences
